# *TLR1* Variant H305L Associated with Protection from Pulmonary Tuberculosis

**DOI:** 10.1371/journal.pone.0156046

**Published:** 2016-05-23

**Authors:** Christian G. Meyer, Norbert Reiling, Christa Ehmen, Gerd Ruge, Ellis Owusu-Dabo, Rolf D. Horstmann, Thorsten Thye

**Affiliations:** 1 Bernhard Nocht Institute for Tropical Medicine, Dept. Molecular Medicine, Hamburg, Germany; 2 Institute for Tropical Medicine, Eberhard-Karls University, Tübingen, Germany; 3 Division of Microbial Interface Biology, Research Center Borstel, Borstel, Germany; 4 Kumasi Centre for Collaborative Research in Tropical Medicine, Kumasi, Ghana; 5 School of Medical Sciences, Dept. of Community Health, Kwame Nkrumah University of Science and Technology, Kumasi, Ghana; University of Birmingham, UNITED KINGDOM

## Abstract

Toll like receptors (TLR) are key elements of the innate immune response and involved in the recognition of pathogens. To test common and rare *TLR* variants involved in susceptibility or resistance to infection with *Mycobacterium tuberculosis* we screened the exons of the genes encoding TLR 1, 2, 4, and the adaptor molecule TIRAP in more than 4500 tuberculosis (TB) cases and controls from Ghana. The analysis yielded 109 variants with possible functional impact, including 101 non-synonymous variants, three stop-variants, and five indels. Association analyses yielded a significant result for the *TLR1* variant rs3923647, conferring strong protection against TB (Odds ratio [OR] 0.21, CI confidence interval [CI] 0.05–0.6, P_*nominal*_ 1 x 10^−3^) when applying a recessive model of inheritance. Replication analyses with an additional 3370 Ghanaian cases and control samples, and with data from a recent TB study of 533 African-Americans confirmed the protective effect and resulted in a combined OR of 0.19, with a nominal P value of 2.2 x 10^−5^, and a corrected P value of 4.1 x 10^−4^. The SNP is located near the binding pocket of TLR1 and causes an amino acid exchange from histidine to leucine at position 305. The observed effect may, therefore, be attributable to structural changes in the recognition site of the TLR1 molecule, allowing to bind those mycobacterial ligands which preferentially may induce a protective immune response. This is supported by the analysis of BCG-stimulated peripheral blood mononuclear cells, showing increased induction of the proinflammatory cytokine IFN-γ in carriers of the mutant TLR1 rs3923647 TT genotype, compared to the IFN-γ levels of individuals with the AT and AA genotypes.

## Introduction

Tuberculosis (TB) remains a global threat with more than one third of the world’s population infected with *Mycobacterium tuberculosis*. The spread of multi-drug resistant *M*. *tuberculosis* strains and the current lack of an efficient vaccine underlines the need for a better understanding of relevant host-pathogen interactions. Factors playing a decisive role in the development of clinically manifest infections include environmental risks, the pathogen´s virulence, and the immune status and genetic architecture of the host [1,2,3,4,5,6]. Variation of genes, including those whose protein products are involved in essential aspects of innate immunity, among them the toll like receptor (TLR) family, has frequently been studied in TB [7,8].

The group of human TLRs consists of ten receptors which are commonly expressed on sentinel cells of the immune system, in particular monocytes and dendritic cells. Distinct binding specificities of each TLR subtype allow to recognize a variety of evolutionarily conserved molecules, addressed as pathogen-associated molecular patterns (PAMPs) [7,8]. PAMPs form a diverse group of molecules, among them immunogenic compounds of pathogens, including LPS, glycolipids, lipoproteins, flagellin, RNA and DNA and others [9]. TLRs can broadly be categorized into two major groups that either sense PAMPs on cell surfaces (TLR 1, -2, -4, and -6) or in the cytosolic compartment (TLRs 3, -5, -7, -8, and -9). Dimerization of TLR chains with binding of PAMPs to the C-terminal leucine-rich repeats initiate TLR signalling. Downstream signal transduction processes are triggered by binding of cytosolic adaptor molecules such as MyD88, TIRAP, and TRIF to the intracellular Toll-interleukin 1 (IL-1) receptor (TIR) domain. The TLR signal cascade, through activation of the transcription factor Nf-κB, induces secretion of proinflammatory cytokines [9].

Innate immune sensors like TLRs are crucial for the recognition of mycobacterial components and in mounting an effective immune response to *M*. *tuberculosis* [10]. In particular the TLRs 1, 2, 4, and 9 are capable of binding a variety of mycobacterial molecules [10]. Genetic variations, in particular those occurring in sequences coding for the binding domains of TLRs, have frequently been analysed in TB case-control studies. Two recent studies have investigated TLR variation in more detail. The exonic region of the *TLRs 1*, *2*, *4*, *6*, and *10* genes were genotyped in three TB study groups comprising of 1,312 individuals [11]. When comparing the occurrence of rare non-synonymous variants between cases and controls associations were found in the groups of African-Americans, European Americans and Hispanics. Associations were also observed with a distinct haplotype consisting of TLR1-248S, TLR1-602I and TLR6-249S among African Americans. However, no consistent association results were identified across the three groups of this study. Velez et al. analysed 71 variants of *TLR 1*, *2*, *4*, *6*, and *9* in African-Americans, Africans from Guinea-Bissau and Caucasians in TB case-control groups [12]. Moderately significant associations were found in two of the three groups analysed with the *TLR9* SNPs rs352143 and rs5743836, and a promoter indel of *TLR2*. Other smaller TB studies mostly analysed single *TLR* variants. Again, no consistent results were obtained so far across different studies with *TLR* variants tested in susceptibility to *M*. *tuberculosis* [13,14].

The aim of our study was to extend the previous investigations by significantly increasing the number of participants to more than 4500 individuals. All non-synonymous variants in the coding regions of the *TLR 1*, *2*, *4* and *TIRAP* genes were identified by high-resolution melting analysis (HRM) and tested for association. The large number of individuals investigated in the present study allowed to analyse the role of rare *TLR* variants in TB in more detail.

## Results

### Genotyping

After screening the exons of *TLRs 1*, *2*, *4*, and *TIRAP* in 4588 samples, HRM analyses revealed 101 non-synonymous variants, 5 insertions or deletions, and 3 variants causing premature stop signals. Fourty-seven of the 109 variants were newly identified (see S1 Table). Ninety-four variants occurred at frequencies < 1% and of those, 50 SNPs were found with a single nucleotide exchange only (Table 1 and S1 Table).

**Table 1:** Number of genetic variants identified.

	all SNPs (n)	non-synonymous SNPs (n)	Insertions / Deletions (n)	Stop SNPs (n)	MAF<1% (n)	MAF≥1% (n)
TLR1	36	34	2	0	28	8
TLR2	25	24	0	1	24	1
TLR4	32	28	2	2	29	3
TIRAP	16	15	1	0	13	3
total	109	101	5	3	94	15

MAF, minor allele frequency

When comparing cases with controls, *TLR1* SNP rs3923647 was, applying Fishers exact test, significantly associated with relative protection from TB (P_nominal_ 0.001). After correction for multiple testing (19 variants tested), an odds ratio (OR) of 0.21 (confidence interval [CI] 0.05–0.6) and a corrected P value of 0.019 was obtained in a recessive model. In order to validate the finding, we tested an additional 324 TB cases and 3046 controls from Ghana, resulting in an OR of 0.33 and a P value of 0.36 (Table 2). A joined analysis comprising the two Ghanaian data sets and data of another TB study in African-Americans of SNP rs3923647 yielded a strong significant result (OR 0.19, CI 0.06–0.48, P_nominal_ 2.2x10^-5^ and P_corrected_ 4.1x10^-4^; recessive model). Analysis of the Hardy-Weinberg equilibrium (HWE) revealed a significant deviation of SNP rs3923647 genotypes in the Ghanaian control group. In order to exclude technical artifacts of the genotyping technique we designed a different set of PCR primers to re-analyse the rs3923647 genotypes and confirmed the initial genotyping results. Notably, rs3923647 HWE assessment in the aforementioned study of African-Americans also yielded significant deviation (Table 2).

**Table 2:** Association results of TLR1 variant rs3923647.

*TLR1* rs3923647 H305L		GT	cases n (frequency)	controls n (frequency)	OR	CI	P	HWE controls P
HRM group	4574	AA	1714(86.0)	2210 (85.6)	1			
		AT	276 (13.8)	345(13.4)	1.03	0.87–1.24		
		TT	4 (0.2)	25 (1.0)	0.21	0.05–0.60	0.001	0.006
Replication group	3370	AA	270 (83.3)	2597 (85.3)	1			
		AT	53 (16.4)	420 (13.8)	1.21	0.87–1.67		
		TT	1 (0.3)	29 (0.9)	0.33	0.01–2.02	0.36	0.01
African American	533	AA	309 (91.2)	175 (90.2)	1			
Ma et al.		AT	30 (8.8)	17 (8.8)	1.00	0.52–1.99		
		TT	0 (0)	2 (1.0)	n.a.	n.a.	0.13	0.04
Joined analysis	8477	AA	2293	4982	1			
		AT	359	782	1.00	0.87–1.14		
		TT	5	56	0.19	0.06–0.48	2.2 x 10^−5^	
						corrected	4.1 x 10^−4^	

P values are calculated using Fishers exact test. P, nominal P value; P_corrected_, P value after correction for multiple testing with a Bonferroni factor of 19. GT, genotype; OR, odds ratio; CI, 95% confidence interval. OR (trend), estimates of an additive genetic model. HWE p, test of deviation from Hardy Weinberg equilibrium

All non-synonymous variants of the genes analysed were subjected to gene-wise rare variant analysis. Three algorithms (Burden-test, EREC-analysis, SKAT-method) did not reveal associations with any combination of rare variants of the four genes studied (Table 3).

**Table 3:** Gene-set associations tests.

	T1	SKAT	EREC
Gene	P	P	P
TLR1	2.4 x 10^−1^	3.4 x 10^−1^	3.7 x 10^−1^
TLR2	8.1 x 10^−1^	5.2 x 10^−1^	8.2 x 10^−1^
TLR4	2.5 x 10^−1^	4.3 x 10^−1^	4.3 x 10^−1^
TIRAP	9.4 x 10^−1^	5.7 x 10^−1^	7.2 x 10^−1^

T1, fixed-threshold test with a MAFs of <1%; SKAT, SNP-set kernel association test; EREC, estimated regression coefficients analysis applying 1 million permutations; MAF, minor allele frequency. The SKAT and EREC test were adjusted for age, sex and affiliation to the Ghanaian ethnic groups.

### Signatures of selection

We tested extended haplotype homozygosity applying the iHS algorithm in a subgroup of 2377 Ghanaian individuals. No strong signs of positive selection (iHS score 1.59) appears to be present in the Ghanaian study group when testing variant rs3923647.

### Interferon gamma production in PBMCs after BCG stimulation

To assess whether H305L exerts any effects on IFN-γ production, we tested IFN-γ mRNA expression in peripheral blood mononuclear cells (PBMCs) from Ghanaian study participants carrying the three genotypes of rs3923647. At least duplicate assays were performed for each genotype. IFN-γ mRNA expression was assessed in PBMC incubated with *M*. *bovis* Bacillus Calmette-Guérin (BCG). Higher IFN-γ mRNA levels of cells from individuals carrying the *TLR1* rs3923647 TT genotype compared to carriers of the AT and AA genotypes were observed (P 0.05, one-tailed T-test). The mean IFN-γ production was measured as cDNA expression ratio of IFN-γ and the household gene HPRT1. Cells of individuals carrying the TT genotype had a ratio of 0.55 compared to a ratio 0.32 of AT and AA carriers.

## Discussion

In this study, we extensively screened for SNPs of *TLR 1*, *2*, *4* and *TIRAP* to analyse the contribution of rare and common variants in TB. The non-synonymous *TLR1* variant H305L (rs3923647) was strongly associated with protection from *M*. *tuberculosis* infection when applying a recessive model of inheritance. Carriers of the TT genotype had a five times decreased risk of developing active TB compared to carriers of the genotypes AA and AT. The protective variant causes a substitution of the hydrophilic histidine with the hydrophobic leucine at position 305 of TLR1. The variant occurs at moderate allele frequencies (8% Ghana, this study; 8% Nigeria according to the 1000 Genomes Project) in these West-African populations.

The contribution of TLR signalling in innate immunity to *M*. *tuberculosis* is not well understood. Several of the nine TLR molecules recognized in humans so far are involved in sensing mycobacterial components. However, recognition of *M*. *tuberculosis* depends mainly on the presence of the TLR1/2 heterodimer, which is able to bind several mycobacterial lipoproteins and glycolipids [15,16,17]. Studies analysing TLR2 deficient mice have revealed an inconclusive picture of either protective or detrimental effects exerted by TLR1/2 signalling in TB. While some studies report an increased TB risk in TLR2 deficient mice [18,19], others have not found differences between TLR2 knock-out and wild-type mice of TB susceptibility [20,21]. After infection with 100 mycobacteria, TLR2 deficient mice were as resistant as were congenic control mice. Granuloma formation, macrophage activation, and secretion of proinflammatory cytokines in response to low-dose aerosol infection were identical in mutant and control mice. However, high-dose aerosol challenge with 2000 CFU *M*. *tuberculosis* revealed that TLR2, but not TLR4 deficient mice were more susceptible than control mice.

**Fig 1:**
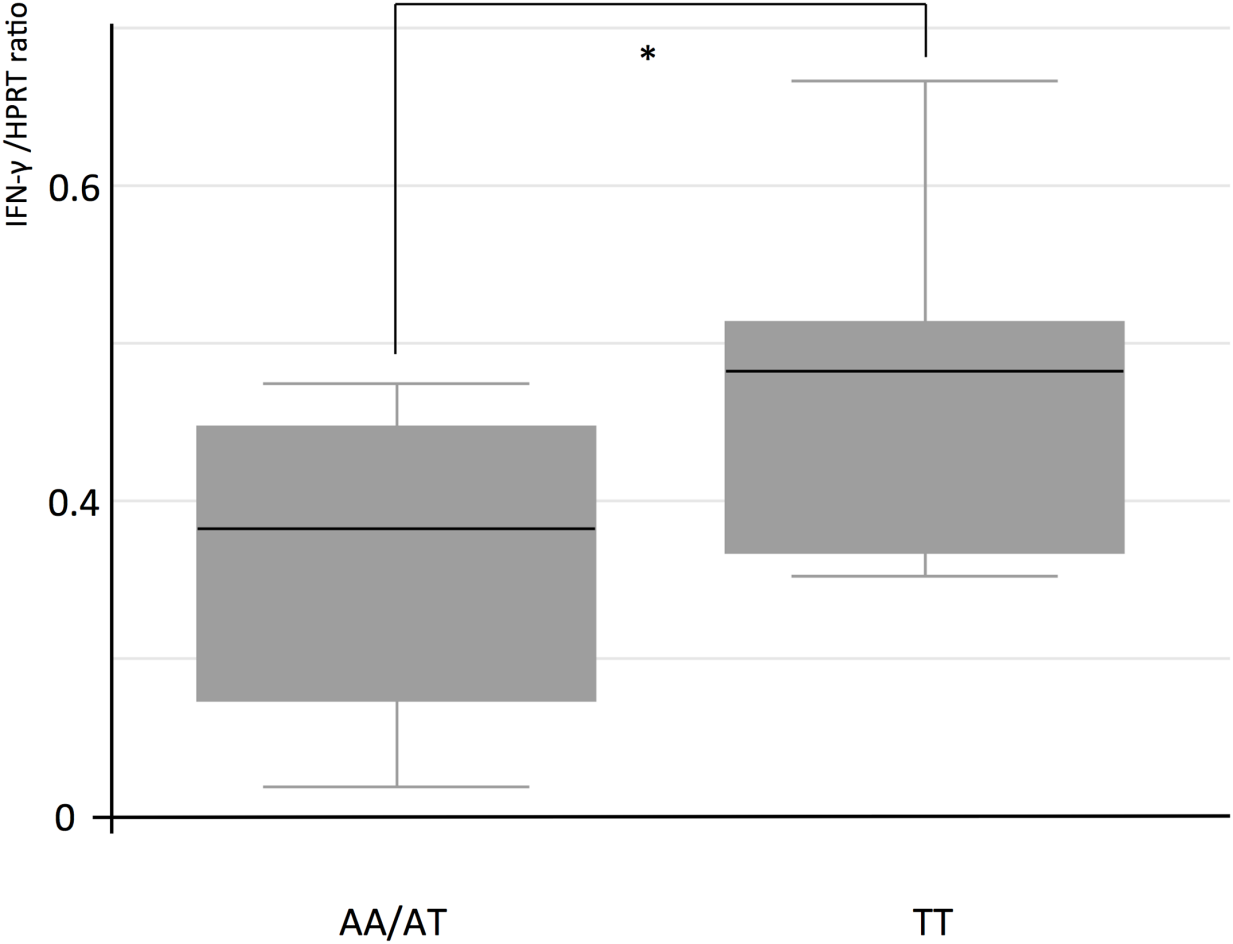
IFN-y expression in *M*. *bovis* BCG stimulated cultured PBMCs. Comparison of IFN-y mRNA expression in *M*. *bovis* BCG stimulated cultured PBMCs of Ghanaian individuals carrying either the TT genotype or the AT/AA genotype of TLR1 SNP rs3923647. Comparison of IFN-y levels yields a significant difference between TT and AT/AA carriers (* P = 0.05, T-test).

TLR1/2 signalling appears to depend on the duration of activation exerted by mycobacterial ligands. It has been shown that prolonged activation of TLR2 may impair the immune response through inhibition of CIITA transactivation, downregulation of MHC II and FcgRI receptors, and to interfere with downstream signalling of the IFN-γ pathway [10]. Mycobacterial agonists of the TLR1/2 heterodimer are mainly triacylated lipopeptides, among them LpqH, LprA, LprG, and PhoS1, and several glycolipids such as lipoarabinomannan, lipomannan, and phosphatidylinositol mannoside [22,23]. Although the underlying mechanism conferring the protective effect that we observed with genotype rs3923647 TT are not clear, we assume that, due to its position close to the ligand-binding site of TLR1, H305L may cause structural changes within the binding pocket, and thus modify the conditions how mycobacterial ligands are recognized by the receptor. Alterations of the protein structure are well conceivable, as predictions determined by the SIFT algorithm of the likely effect of variant rs3923647 on the protein function revealed a potentially deleterious effect (score = 0). Thus, distinct mycobacterial lipoprotein or glycolipid ligands may exhibit different binding properties when carrying either the mutant or the wild type TLR1/2 receptor. Mycobacterial ligands which can impair TLR signalling could have a decreased ability to bind to mutant receptors, allowing only mycobacterial ligands that can lead to a protective immune response to bind effectively. Supporting evidence was reported in a study where the inflammatory response of PBMCs from BCG-vaccinated newborns in South Africa, depending on their rs3923647 genotypes, was investigated [24]. IFN-γ levels were in that study five times higher in rs3923647 TT carriers after BCG stimulation, compared to carriers of the AT and AA genotypes. A similar effect could in the present study be detected with PBMCs isolated from individuals of the Ghanaian study group stimulated with BCG (Fig 1). Following TLR1/2 activation, increased IFN-γ levels of the mutant receptor as observed by us and in the study from South Africa may explain to some extent the protective effect exerted by the variant H305L.

Signatures of positive selection were recently identified at the *TLR1* locus by analyses of extended haplotype homozygosity [25]. The strong protective effect that we found in the association analysis allows to assume that selection might have acted on the *TLR1* SNP rs3923647 in our study group. However, no signs of selection were identified when calculating the integrated haplotype score (iHS) with variant rs3923647. This may be attributable to the fact that selection acts very slowly over time on rare advantageous recessive variants such as rs3923647. In contrast, the significant deviations from HWE observed might nevertheless indicate natural selection acting in the present generation of the Ghanaian study population, favouring the accumulation of the rs3923647 TT genotype in the the TB control groups. Notably, data available from the 1000 Genomes Project show also deviation from HWE for the *TLR1* genotypes under study (TT 2365, AT 132, AA 7; P = 0.0006; http://browser.1000genomes.org/).

In conclusion, we have identified a variant, *TLR1* H305L, that strongly confers protection from pulmonary TB. The variant is located in the binding domain of the TLR1 receptor and has a strong potential to alter the binding structure of the receptor. However, further functional studies are necessary to fully elucidate the role that TLR1 H305L plays in the host-pathogen interaction.

## Material and Methods

### Study group

The study was performed with DNA samples from participants of an association study on susceptibility and resistance to pulmonary TB in Ghana enrolled between 2001 and 2004 at the Korle Bu Teaching Hospital in Accra, the Komfo Anokye Teaching Hospital in Kumasi and additional urban and district hospitals as previously described in detail [26]. In brief, the study group consisted of 1999 HIV-negative individuals with smear-/culture-positive pulmonary TB and 2589 healthy controls. Cases and controls belonged to the ethnic groups of Akan, Ga-Adangbe, Ewe, and minor ethnic groups from northern Ghana.

Phenotyping of patients was based on the medical histories and self-reporting of symptoms, physical examination, chest X-ray, HIV-1/2 testing, Ziehl-Neelsen staining of sputum smears and culturing of *M*. *tuberculosis* on Loewenstein-Jensen agar [27].

The study design and all protocols were approved by the Committee on Human Research, Publications and Ethics, School of Medical Sciences, Kwame Nkrumah University, Kumasi, Ghana, and the Ethics Committee of the Ghana Health Service, Accra. Informed consent was procured by signature or thumbprint after a detailed explanation of the study aims. Medical data and samples from patients were analyzed anonymously.

### Genetic screening

We subjected 4588 DNA samples to *TLR1*, *2*, *4* and *TIRAP* variant screening by high-resolution melting (HRM) analysis on a LightCycler^®^ 480 device (Roche Diagnostics, Mannheim, Germany). The aim was to identify all exonic, including so far unrecognized, variants of these genes. HRM was applied according to the recommendations of the manufacturer. HRM PCR primers and amplicon sizes will be made available upon request. To validate HRM results indicating so far unrecognized variants, independent sequencing assays were carried out to confirm the presence of variants.

### Statistical analysis

Statistical analyses were performed with PLINK 1.07 [28], SCORE-Seq 5.2 [29] and STATA v10.1. (STATA Corporation, College Station, TX, USA). Logistic regression analyses adjusted for age, ethnicity and gender were calculated for single variants of the *TLR* and *TIRAP* genes with a minor allele frequency (MAF) > 1%. Fisher´s exact test was used for variants where cell counts of contingency tables were equal or less than 5. Additive, dominant and recessive modes were tested. We performed corrections for multiple testing for single variants by multiplying P values with the number of SNPs with a MAF > 1%. For variants exhibiting a pairwise linkage disequilibrium of r^2^ > 0.9, we considered only one variant for multiple testing correction. All rare variants of genes with MAFs < 1% were summarized as a one SNP and, thus, as one correction unit, leading to a total of 19 correction units to be considered in the present study.

In an additional analysis we grouped rare *TLR* and *TIRAP* variants, as testing these variants individually would considerably reduce the power to identify significant signals of rare variants. Gene-based association tests were performed with the “SCORE-Seq” software to calculate SNP-set level P values. The analyses were restricted to non-synonymous variants with MAFs less than or equal to 1%, applying a pooling method with a fixed threshold of 1% (T1) for the rare non-synonymous variants of the *TLR1*, *2*, *4* and *TIRAP* genes. The fixed threshold test assumes effects of tested variants to be in the same direction. In addition, we tested two more recently developed methods, which account for combinations of phenotypic effects of protective, neutral and damaging variants. Both methods, the “Sequence Kernel Association Test” (SKAT) [30], and the Estimated Regression Coefficients Analysis (EREC) [29] allow to include covariates into the analysis.

### Selection testing and HWE analyses

To test for selection at the *TLR1* locus we generated haplotypes of 46147 SNPs of chromosome 4 in 2377 individuals of the Ghanaian study group. All non-synonymous *TLR1* SNPs identified by HRM analysis were included. Only SNPs with a minor allele frequency of > 5% were selected for haplotype formation applying the SHAPEIT v2.r727 software (https://mathgen.stats.ox.ac.uk/genetics_software/shapeit/shapeit.html). The integrated haplotype score (iHS) were analysed with the HAPBIN software (https://github.com/evotools/hapbin). iHS values larger than 2 were regarded as putative signatures of selection at the analysed genomic region. To assess potential deviations from Hardy Weinberg equilibrium among controls PLINK was applied.

### IFN-γ mRNA expression

PBMCs of 15 individuals with each 5 of them carrying one of the three genotypes of rs3923647 were isolated from whole blood separated by Ficoll gradient centrifugation. IFN-γ mRNA expression was assessed in PBMCs (1.5 x 10^5^ / 200μl) harvested after 24 hours incubation with sonicated *Mycobacterium bovis* BCG bacteria (10μl of 2.4 x 10^−8^ bacteria/ml). *M*. *bovis* BCG aliquots (2.4 x 10^8^ /ml) were thawed and centrifuged at 4°C, 2250 x g for 10 min. The supernatant was discarded, and the bacteria were resuspended in 1 ml PBS, supplemented with proteinase inhibitor. Bacteria were sonicated for 20 min in closed caps (Branson Sonifier 450 II Classic; Heinemann, Schwäbisch-Gmünd, Germany). Experiments were performed at least in duplicates for cells harbouring the different genotypes. Total RNA was isolated from PBMC using Direct-zol^™^ RNA MiniPrep (Zymo Research, Irvine, USA). For reverse transcription the Maxima^®^ First Strand cDNA Synthesis Kit for RT-qPCR (Fermentas, St-Leon-Rot, Germany) was used. Gene-specific primer pairs (IFN-gamma: Fwd: 5´- GGCATTTTGAAGAATTGGAAAG-3´, Rev: 5´-TTTGGATGCTCTGGTCATCTT-3´; HPRT: 5´-TGACCTTGATTTATTTTGCATACC-3´, Rev:5´- CGAGCAAGACGTTCAGTCCT-3´) and TaqMan^®^ probes (Universal Probe Library, Roche Applied Science, IFNg: #21; HPRT: #73) were designed with the UPL assay design center. RT-qPCR was performed using the LightCycler^®^ 480 Probe Master Kit and the LightCycler^®^ 480 II system (Roche Applied Science). *Cp* values of IFN-gamma and reference gene (HPRT) were determined by the second derivative maximum method. Relative gene expression was calculated considering the individual efficiency of each PCR reaction setup determined by a standard curve.

## Supporting Information

S1 Table Coding variants of *TLR 1*, *2*, *4* and *TIRAP* identified by HRM analysis of 4500 Ghanaian individuals. (DOCX)
