# Supplementary material for: TLR1 Variant H305L Associated with Protection from Pulmonary Tuberculosis
Source: PLoS One. 2016 May 23;11(5):e0156046. doi: 10.1371/journal.pone.0156046 (PMC4877073; doi:10.1371/journal.pone.0156046)
Supplement: S1 Table — (DOCX) [file pone.0156046.s001.docx]

**Supplementary Table 1** Coding variants of *TLR 1*, *2*, *4* and *TIRAP* identified by HRM analysis of 4500 Ghanaian individuals

| GENE | CHR | POS | AA | #rs | CASES | CONTROLS | P | ALLELES | MAF |
| --- | --- | --- | --- | --- | --- | --- | --- | --- | --- |
| TLR1 | 4 | 38798160 | R765C | rs542161166 | 0/0/1999 | 0/1/2588 | 1 | G>A | 0.0001 |
| TLR1 | 4 | 38798210 | S748N | new | 0/1/1998 | 0/0/2589 | 0.44 | C>T | 0.0001 |
| TLR1 | 4 | 38798255 | P733L | rs5743621 | 0/100/1893 | 0/172/2411 | 0.02 | G>A | 0.0297 |
| TLR1 | 4 | 38798403 | I684del | new | 0/1/1998 | 0/2/2587 | 1 | GAT>- | 0.0003 |
| TLR1 | 4 | 38798433 | V674I | rs137853175 | 0/10/1989 | 0/26/2563 | 0.06 | C>T | 0.0039 |
| TLR1 | 4 | 38798561 | L631R | rs5743619 | 0/0/1999 | 0/1/2588 | 1 | A>C | 0.0001 |
| TLR1 | 4 | 38798592 | R621C | rs369737596 | 0/0/1999 | 0/1/2588 | 1 | G>A | 0.0001 |
| TLR1 | 4 | 38798594 | R620Q | rs201366752 | 0/0/1999 | 0/1/2588 | 1 | C>T | 0.0001 |
| TLR1 | 4 | 38798648 | S602I | rs5743618 | 1/55/1943 | 0/56/2532 | 0.17 | C>A | 0.0123 |
| TLR1 | 4 | 38798657 | S599F | rs143576765 | 2/16/1981 | 0/33/2555 | 0.07 | G>A | 0.0058 |
| TLR1 | 4 | 38798693 | V587G | rs5743617 | 3/168/1825 | 5/215/2368 | 0.98 | A>C | 0.0435 |
| TLR1 | 4 | 38798702 | V584A | rs150358789 | 0/0/1999 | 0/1/2588 | 1 | A>G | 0.0001 |
| TLR1 | 4 | 38798777 | P559Q | rs568135901 | 0/1/1998 | 0/0/2589 | 0.44 | G>T | 0.0001 |
| TLR1 | 4 | 38798813 | L547S | new | 0/1/1998 | 0/1/2588 | 1 | A>G | 0.0002 |
| TLR1 | 4 | 38798822 | S544N | rs771697062 | 0/0/1999 | 0/1/2588 | 1 | C>T | 0.0001 |
| TLR1 | 4 | 38798828 | V542A | rs137853172 | 0/24/1975 | 0/33/2556 | 0.89 | A>G | 0.0062 |
| TLR1 | 4 | 38798862 | E531K | new | 0/1/1998 | 0/0/2589 | 0.44 | C>T | 0.0001 |
| TLR1 | 4 | 38799225 | I410V | rs139333505 | 0/2/1997 | 0/0/2589 | 0.19 | C>T | 0.0002 |
| TLR1 | 4 | 38799242 | D404V | new | 0/0/1999 | 0/1/2588 | 1 | T>A | 0.0001 |
| TLR1 | 4 | 38799294 | L387I | new | 0/1/1998 | 0/0/2589 | 0.44 | G>T | 0.0001 |
| TLR1 | 4 | 38799399 | H352N | rs76796448 | 12/272/1710 | 15/305/2254 | 0.19 | G>T | 0.0691 |
| TLR1 | 4 | 38799444 | R337C | rs200457447 | 0/1/1998 | 0/0/2589 | 0.44 | G>A | 0.0001 |
| TLR1 | 4 | 38799446 | T336I | new | 0/0/1999 | 0/1/2588 | 1 | G>A | 0.0001 |
| TLR1 | 4 | 38799449 | G335D | rs141091142 | 0/1/1998 | 0/1/2588 | 1 | C>T | 0.0002 |
| TLR1 | 4 | 38799509 | P315L | rs5743613 | 1/64/1929 | 0/74/2501 | 0.43 | G>A | 0.0153 |
| TLR1 | 4 | 38799524 | D310V | new | 0/1/1998 | 0/0/2589 | 0.44 | T>A | 0.0001 |
| TLR1 | 4 | 38799539 | H305L | rs3923647 | 4/276/1714 | 25/345/2210 | 0.003 | T>A | 0.0735 |
| TLR1 | 4 | 38799545 | S303F | new | 0/0/1999 | 0/1/2588 | 1 | G>A | 0.0001 |
| TLR1 | 4 | 38799710 | N248S | rs4833095 | 18/404/1571 | 29/482/2063 | 0.34 | T>C | 0.1073 |
| TLR1 | 4 | 38799724 | N243fs | new | 0/1/1998 | 0/1/2588 | 1 | A>- | 0.0002 |
| TLR1 | 4 | 38799740 | A238V | rs759842459 | 0/0/1999 | 0/1/2588 | 1 | G>A | 0.0001 |
| TLR1 | 4 | 38799978 | I159V | new | 0/0/1999 | 0/1/2588 | 1 | T>C | 0.0001 |
| TLR1 | 4 | 38800060 | I131M | new | 0/2/1997 | 0/1/2588 | 0.58 | T>C | 0.0003 |
| TLR1 | 4 | 38800101 | H118Y | rs5743612 | 23/352/1592 | 17/454/2078 | 0.21 | G>A | 0.0981 |
| TLR1 | 4 | 38800282 | I57M | rs145135062 | 0/1/1998 | 0/0/2589 | 0.44 | T>C | 0.0001 |
| TLR1 | 4 | 38800403 | I17N | rs147564337 | 0/3/1996 | 0/3/2586 | 1 | A>T | 0.0007 |
| TLR2 | 4 | 154624108 | L17V | rs759023240 | 0/1/1997 | 0/1/2588 | 1 | C>G | 0.0002 |
| TLR2 | 4 | 154624124 | S22F | new | 0/0/1998 | 0/1/2588 | 1 | C>T | 0.0001 |
| TLR2 | 4 | 154624136 | A26V | rs145391095 | 0/34/1964 | 0/38/2551 | 0.55 | C>T | 0.0078 |
| TLR2 | 4 | 154624170 | K37N | new | 0/0/1998 | 0/2/2587 | 0.51 | G>C | 0.0002 |
| TLR2 | 4 | 154624269 | S70R | new | 0/0/1998 | 0/1/2588 | 1 | T>A | 0.0001 |
| TLR2 | 4 | 154624316 | N86S | rs142041844 | 0/0/1998 | 0/2/2587 | 0.51 | A>G | 0.0002 |
| TLR2 | 4 | 154624481 | E141G | new | 0/1/1997 | 0/0/2589 | 0.44 | A>G | 0.0001 |
| TLR2 | 4 | 154624483 | T142A | rs760867997 | 0/2/1996 | 0/3/2586 | 1 | A>G | 0.0005 |
| TLR2 | 4 | 154624501 | L148F | new | 0/1/1997 | 0/0/2589 | 0.44 | C>T | 0.0001 |
| TLR2 | 4 | 154624513 | Q152Ter | new | 0/2/1996 | 0/0/2589 | 0.1897 | C>T | 0.0002 |
| TLR2 | 4 | 154624648 | I197F | rs144030660 | 0/10/1988 | 0/19/2570 | 0.3535 | A>T | 0.0032 |
| TLR2 | 4 | 154624669 | I204V | new | 0/0/1998 | 0/1/2588 | 1 | A>G | 0.0001 |
| TLR2 | 4 | 154625422 | I455V | new | 0/0/1998 | 0/1/2588 | 1 | A>G | 0.0001 |
| TLR2 | 4 | 154625456 | N466S | new | 0/1/1997 | 0/0/2589 | 0.44 | A>G | 0.0001 |
| TLR2 | 4 | 154625771 | R571H | rs61735277 | 0/44/1946 | 1/43/2534 | 0.23 | G>A | 0.0097 |
| TLR2 | 4 | 154625795 | R579H | rs5743703 | 0/33/1943 | 0/58/2502 | 0.17 | G>A | 0.0100 |
| TLR2 | 4 | 154625864 | I602N | new | 0/0/1998 | 0/1/2588 | 1 | T>A | 0.0001 |
| TLR2 | 4 | 154626008 | R650Q | rs200483398 | 0/0/1998 | 0/1/2588 | 1 | G>A | 0.0001 |
| TLR2 | 4 | 154626043 | E662K | new | 0/1/1997 | 0/1/2588 | 1 | G>A | 0.0002 |
| TLR2 | 4 | 154626062 | P668R | new | 0/0/1998 | 0/1/2588 | 1 | C>G | 0.0001 |
| TLR2 | 4 | 154626150 | H697Q | rs752887829 | 0/2/1996 | 0/1/2588 | 0.58 | C>A | 0.0003 |
| TLR2 | 4 | 154626271 | E738Q | rs142286429 | 0/10/1988 | 0/19/2570 | 0.35 | G>C | 0.0032 |
| TLR2 | 4 | 154626335 | K759M | new | 0/0/1998 | 0/1/2588 | 1 | A>T | 0.0001 |
| TLR2 | 4 | 154626355 | M766V | rs182578104 | 0/4/1994 | 0/6/2583 | 1 | A>G | 0.0011 |
| TLR2 | 4 | 154626368 | Q770R | new | 0/8/1990 | 0/7/2582 | 0.45 | A>G | 0.0016 |
| TLR4 | 9 | 120466830 | E27G | new | 0/1/1998 | 0/0/2589 | 0.44 | A>G | 0.0001 |
| TLR4 | 9 | 120470895 | D50N | rs776561489 | 0/0/1999 | 0/1/2588 | 1 | G>A | 0.0001 |
| TLR4 | 9 | 120470900 | N51K | rs148151027 | 0/0/1999 | 0/2/2587 | 0.51 | C>A | 0.0002 |
| TLR4 | 9 | 120474693 | G96V | new | 0/0/1999 | 0/1/2588 | 1 | G>T | 0.0001 |
| TLR4 | 9 | 120474762 | L119R | new | 0/1/1998 | 0/1/2588 | 1 | T>G | 0.0002 |
| TLR4 | 9 | 120474969 | Q188R | new | 0/11/1988 | 0/13/2576 | 0.84 | A>T | 0.0026 |
| TLR4 | 9 | 120475143 | C246S | rs5030714 | 0/3/1996 | 0/1/2588 | 0.32 | G>C | 0.0004 |
| TLR4 | 9 | 120475206 | G267E | new | 0/1/1998 | 0/0/2589 | 0.44 | G>A | 0.0001 |
| TLR4 | 9 | 120475266 | E287G | rs76014534 | 0/1/1998 | 0/1/2588 | 1 | A>G | 0.0002 |
| TLR4 | 9 | 120475271 | R289Ter | new | 0/1/1998 | 0/0/2589 | 0.44 | C>T | 0.0001 |
| TLR4 | 9 | 120475298 | D298N | rs145284280 | 0/0/1999 | 0/1/2588 | 1 | G>A | 0.0001 |
| TLR4 | 9 | 120475302 | D299G | rs4986790 | 26/389/1566 | 17/480/2061 | 0.06 | A>G | 0.1052 |
| TLR4 | 9 | 120475547 | S381R | new | 0/0/1999 | 0/2/2587 | 0.51 | A>G | 0.0002 |
| TLR4 | 9 | 120475561 | L385F | rs11536884 | 0/27/1952 | 0/37/2510 | 0.90 | G>T | 0.0071 |
| TLR4 | 9 | 120475602 | T399I | rs4986791 | 1/42/1907 | 0/42/2509 | 0.18 | C>T | 0.0096 |
| TLR4 | 9 | 120475826 | E474K | rs5030718 | 5/168/1811 | 4/185/2376 | 0.21 | G>A | 0.0408 |
| TLR4 | 9 | 120475936 | Q510H | rs5030719 | 3/155/1838 | 4/180/2396 | 0.56 | G>T | 0.0381 |
| TLR4 | 9 | 120475983 | N526T | new | 0/0/1999 | 0/1/2588 | 1 | A>C | 0.0001 |
| TLR4 | 9 | 120476016 | T537M | rs151068160 | 0/1/1998 | 0/0/2589 | 0.44 | C>T | 0.0001 |
| TLR4 | 9 | 120476090 | Q562fs | new | 0/1/1998 | 0/1/2588 | 1 | ->A | 0.0002 |
| TLR4 | 9 | 120476382 | M659T | rs140816474 | 0/1/1998 | 0/3/2586 | 0.64 | T>C | 0.0004 |
| TLR4 | 9 | 120476415 | G670D | rs200829727 | 0/1/1998 | 0/0/2589 | 0.44 | G>A | 0.0001 |
| TLR4 | 9 | 120476456 | D684Y | rs148187542 | 0/0/1999 | 0/1/2588 | 1 | G>T | 0.0001 |
| TLR4 | 9 | 120476576 | H724D | new | 0/0/1999 | 0/2/2587 | 0.51 | C>G | 0.0002 |
| TLR4 | 9 | 120476639 | R745C | rs199632399 | 0/0/1999 | 0/1/2588 | 1 | C>T | 0.0001 |
| TLR4 | 9 | 120476693 | R763S | new | 0/0/1999 | 0/1/2588 | 1 | C>T | 0.0001 |
| TLR4 | 9 | 120476772 | L789R | new | 0/1/1998 | 0/0/2589 | 0.44 | T>G | 0.0001 |
| TLR4 | 9 | 120476798 | E798K | rs200020265 | 0/1/1998 | 0/0/2589 | 0.44 | G>A | 0.0001 |
| TLR4 | 9 | 120476816 | R804W | rs55786277 | 0/2/1997 | 0/0/2589 | 0.19 | C>T | 0.0002 |
| TLR4 | 9 | 120476817 | R804Q | rs149989546 | 0/9/1990 | 0/9/2580 | 0.64 | G>A | 0.0020 |
| TLR4 | 9 | 120476834 | R810Ter | rs199923181 | 0/1/1998 | 0/0/2589 | 0.44 | C>T | 0.0001 |
| TLR4 | 9 | 120476890 | T829fs | new | 0/1/1998 | 0/1/2588 | 1 | TACAGG>- | 0.0002 |
| TIRAP | 11 | 126160814 | A9P | rs8177369 | 0/73/1870 | 0/88/2448 | 0.63 | G>C | 0.0180 |
| TIRAP | 11 | 126162468 | S55N | rs3802813 | 21/220/1749 | 14/299/2268 | 0.13 | G>A | 0.0644 |
| TIRAP | 11 | 126162479 | P59S | new | 0/0/1978 | 0/2/2555 | 0.51 | C>T | 0.0002 |
| TIRAP | 11 | 126162480 | P59R | new | 0/1/1977 | 0/0/2557 | 0.44 | C>G | 0.0001 |
| TIRAP | 11 | 126162550 | W82C | new | 0/1/1976 | 0/0/2565 | 0.44 | G>C | 0.0001 |
| TIRAP | 11 | 126162552 | S83N | new | 0/0/1977 | 0/2/2563 | 0.51 | G>A | 0.0002 |
| TIRAP | 11 | 126162614 | V104F | new | 0/7/1970 | 0/4/2561 | 0.23 | G>T | 0.0012 |
| TIRAP | 11 | 126162636 | T111fs | new | 0/0/1977 | 0/1/2564 | 1 | C>- | 0.0001 |
| TIRAP | 11 | 126162704 | C134R | rs74937157 | 0/6/1971 | 0/13/2552 | 0.36 | T>C | 0.0021 |
| TIRAP | 11 | 126162731 | R143W | rs200632029 | 0/1/1976 | 0/0/2565 | 0.44 | C>T | 0.0001 |
| TIRAP | 11 | 126162732 | R143L | rs199917692 | 0/1/1976 | 0/0/2565 | 0.44 | G>T | 0.0001 |
| TIRAP | 11 | 126162747 | T148K | new | 0/1/1976 | 0/0/2565 | 0.44 | C>A | 0.0001 |
| TIRAP | 11 | 126162815 | A171S | rs141792148 | 0/4/1986 | 0/7/2568 | 0.77 | G>T | 0.0012 |
| TIRAP | 11 | 126162832 | I176M | rs368918609 | 0/6/1984 | 0/6/2569 | 0.77 | C>G | 0.0013 |
| TIRAP | 11 | 126162843 | S180L | rs8177374 | 0/5/1985 | 0/7/2568 | 1 | C>T | 0.0013 |
| TIRAP | 11 | 126162893 | V197I | rs7932976 | 1/105/1888 | 2/174/2411 | 0.082 | G>A | 0.0311 |

HRM, high-resolution melting point analysis; CHR, chromosome; POS, positions of variants referring to the chromosomal build NC_000004.11 for *TLR1* and *TLR2*, to build NC_000009.11 for *TLR4*, and to build NC_0000011.9 for the *TIRAP* gene; AA, amino acid exchange; CASES/CONTROLS, numbers of each of the three genotypes per SNP; P, P values obtained by calulcating the genotypic association test between cases and controls; MAF, minor allele frequency.
